# Patterns of use of biological and genetic markers for chronic lymphocytic leukemia and acute myeloid leukemia in Puerto Rico

**DOI:** 10.1002/cam4.5482

**Published:** 2022-11-25

**Authors:** Karen J. Ortiz‐Ortiz, Carlos R. Torres‐Cintrón, Tonatiuh Suárez Ramos, Maira A. Castañeda‐Avila, Luis A. Cotto Santana, Guillermo Tortolero‐Luna

**Affiliations:** ^1^ Division of Cancer Control and Population Sciences University of Puerto Rico Comprehensive Cancer Center San Juan Puerto Rico; ^2^ Puerto Rico Central Cancer Registry University of Puerto Rico, Comprehensive Cancer Center San Juan Puerto Rico; ^3^ Department of Health Services Administration, Graduate School of Public Health, Medical Sciences Campus University of Puerto Rico San Juan Puerto Rico; ^4^ Department of Population and Quantitative Health Sciences University of Massachusetts Chan Medical School Worcester Massachusetts USA; ^5^ Auxilio Mutuo Hospital San Juan Puerto Rico

**Keywords:** acute myeloid leukemia (AML); genetic markers, chronic lymphocytic leukemia (CLL), Hispanics, Puerto Rico

## Abstract

**Background:**

The use of markers has stimulated the development of more appropriate targeted therapies for chronic lymphocytic leukemia (CLL) and acute myeloid leukemia (AML). We assessed the use and prevalence of biological and genetic markers of CLL and AML in the homogeneous Hispanic population of Puerto Rico.

**Methods:**

We used the Puerto Rico CLL/AML Population‐Based Registry, which combines information from linked databases. Logistic regression models were used to examine factors associated with biological and genetic testing.

**Results:**

A total of 926 patients 18 years or older diagnosed with CLL (*n* = 518) and AML (*n* = 408) during 2011–2015 were included in this analysis. Cytogenetic testing (FISH) was reported for 441 (85.1%) of the CLL patients; of those, 24.0% had the presence of trisomy 12, 9.5% carried deletion 11q, 50.3% carried deletion 13q, and 6.3% carried deletion 17p. Regarding AML, patients with cytogenetics and molecular tests were considered to determine the risk category (254 patients), of which 39.8% showed poor or adverse risk. Older age and having more comorbidities among patients with CLL were associated with a lower likelihood of receiving a FISH test.

**Conclusions:**

Although prognostic genetic testing is required for treatment decisions, the amount of testing in this Hispanic cohort is far from ideal. Furthermore, some tests were not homogeneously distributed in the population, which requires further exploration and monitoring. This study contributes to the field by informing the medical community about the use and prevalence of biological and genetic markers of CLL and AML. Similarly, it has the potential to improve the management of CLL and AML through benchmarking.

## Graphical abstract

We assessed the use and prevalence of biological and genetic markers of chronic lymphocytic leukemia (CLL) and acute myeloid leukemia (AML) in the homogeneous Hispanic population of Puerto Rico. In Puerto Rico, these tests are not performed consistently among patients with CLL and AML; however, the frequency of genetic testing was higher than that reported in other studies.

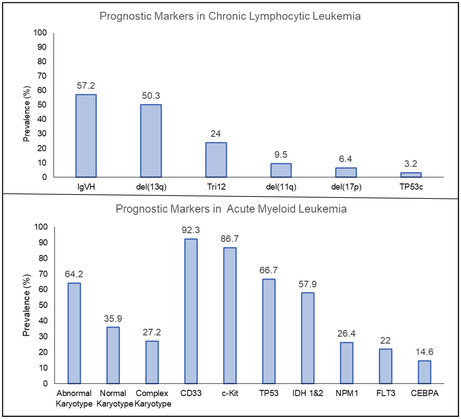

## 1 INTRODUCTION

Worldwide, leukemia remains one of the leading causes of cancer morbidity and mortality. Leukemia is subdivided into myeloid or lymphoid cells, depending on the starting location. Chronic lymphocytic leukemia (CLL) and acute myeloid leukemia (AML) are the most frequent types of leukemia among the elderly population.^1^ Given the heterogeneity of CLL, some patients may live many years after diagnosis without the need for therapy, while others die within the first year from disease‐related complications.^1^ AML is characterized by a group of phenotypic and genetically heterogeneous hematologic diseases, categorized by the clonal expansion of myeloid precursor with decreased differentiation capacity.^2^

In Puerto Rico, leukemia is the ninth most common cancer, with an incidence rate of 10.2 per 100,000 population, and the eighth leading cause of cancer‐related death, with a mortality rate of 4.1 per 100,000 population.^3^ When stratified by subtype, AML and CLL are the most frequently diagnosed types of leukemia, with an age‐adjusted incidence rate of 3.1 and 2.6, respectively. The Commonwealth of Puerto Rico is the largest US territory, with over 3.2 million population.^4^ Puerto Ricans represent the second largest Hispanic population in the country, with more than 4 million living in the continental United States. Nearly 99% of the population living in Puerto Rico identify themselves as Hispanics.^4^ Puerto Rico's population is older than the continental United States, with about 21.3% of the Puerto Rican population 65 years or older.^4^ Puerto Rico faces a significant demographic shift due to migration to the continental United States and low fertility rates.^5^ In Puerto Rico, more than 92% of the population is covered by health insurance, and most receive Medicaid or Medicare (60%).^4^ Nearly 31.5% of Puerto Rico's population has private health insurance, including employer‐sponsored plans and plans purchased directly from insurers. Insurance companies cover cancer diagnostic procedures, including genetic testing, although patients could be responsible for out‐of‐pocket expenses depending on insurance coverage.

During the past decades, novel biomarkers have changed the way physicians treat patients with leukemia and assign targeted therapies. The use of markers in patients with CLL has provided important information on the prognosis of the disease and has stimulated the development of more appropriate targeted therapies.^6^ Some of the most reliable molecular prognostic markers offered in routine diagnostic tests are the mutational status of the immunoglobulin heavy chain variable (IGHV) gene and those detected by the fluorescence in situ hybridization technique (FISH).^7^ For AML, there are cytogenetic alterations producing fusion genes that encode aberrant proteins with altered functional characteristics. Polymerase chain reaction (PCR) test is recommended to detect leukemic cells during and after treatment because it has the highest analytic sensitivity.^8^ Depending on the results of the chromosome tests, patients with AML are stratified into three categories that help to determine their prognosis and response to treatment.^9^

The cytogenetic analysis of AML and CLL has become essential for the diagnosis, classification, prognostic stratification, and treatment guidance of the disease.^9^, ^10^, ^11^, ^12^ However, like most population‐based registries, the Puerto Rico Central Cancer Registry (PRCCR) does not collect extensive clinical information or cancer‐related biological and genetic markers, limiting the use of these registries to address critical research questions. Nevertheless, data from population‐based registries can be linked to different databases to expand the number of variables collected and increase their potential to address these critical research questions. To our best knowledge, no study has evaluated the use of these prognostic factors for CLL or AML in Puerto Rico, an aging Hispanic population. Therefore, in partnership with an external entity, we created the Puerto Rico CLL/AML Population‐Based Registry, which leverages the PRCCR capabilities to assess the pattern of use and prevalence of biological and genetic markers for CLL and AML and examine the factors associated with the administration of biological and genetic tests.

## 2 METHODS

### 2.1 Data sources

The PRCCR, one of the oldest population‐based cancer registries in the world, is responsible for collecting, analyzing, and publishing information on all cancer cases diagnosed and/or treated among residents of Puerto Rico. Since 1997, the PRCCR has been part of the Centers for Disease Control and Prevention's National Program of Cancer Registries and uses the Surveillance, Epidemiology, and End Results (SEER) program and the North American Association of Central Cancer Registries (NAACCR) standards for coding data. Furthermore, the PRCCR requests information from hospitals, outpatient clinics, pathology laboratories, and radiotherapy/chemotherapy sites throughout Puerto Rico. Over the years, the PRCCR improved data collection on cancer cases through electronic reporting, achieving complete information on more than 95% of cases since 2010. Additionally, the PRCCR–Health Insurance Linkage Database (PRCCR‐HILD) links the PRCCR database to Medicaid, Medicare, and private insurance data for residents of Puerto Rico and provides information about treatment, medical procedures, comorbidities, costs, and providers. This linkage has allowed us to conduct cancer care delivery research to better understand the patterns of cancer care on the island.

### 2.2 Creation of the Puerto Rico CLL/AML population‐based registry

The PRCCR integrated a multidisciplinary team of oncologists, tumor registrars, epidemiologists, biostatisticians, and informatics to develop the Puerto Rico CLL/AML Population‐Based Registry software and database. The CLL/AML registry uses the PRCCR data and expands the number of clinical, biological, and genetic variables that are not collected regularly. After several meetings with experts, we determined the genetic markers, prognostic factors, laboratory tests, and treatment modalities needed for the project. We took advantage of pathology laboratories that report electronically using *PathPlus*, a PRCCR in‐house software with comprehensive case‐finding protocols to identify incident cases. An infrastructure with extensive algorithms was developed to search for specific CLL and AML‐related biomarkers in pathology reports. A solution was created in Visual Studio to manage variables related to CLL/AML, integrating data from PRCCR's cancer database, Pathology Reports database, electronic medical records (EMR), and PRCCR‐HILD. Furthermore, the Puerto Rico CLL/AML Population‐Based Registry database collected treatment, healthcare utilization, health insurance type, and a modified Charlson's comorbidity index described by Klabunde et al.^13^, ^14^ (see the CLL/AML Management System in Appendix 1). The oncologist trained a qualified tumor registrar to retrieve the variables of interest. Furthermore, the tumor registrar performed a quality control process to ensure the completeness of the diagnosis, tumor markers, and treatment information for the cases of CLL and AML.

### 2.3 Selection criteria

The study population consisted of cases reported to the PRCCR between January 1, 2011, and December 31, 2015, with a diagnosis of CLL (9823) and AML (9840, 9861, 9865–9867, 9869, 9871–9874, 9895–9897, 9898, 9910–9911, 9920), as defined by the *International Classification of Diseases for Oncology, third edition* (ICD‐O‐3). We also excluded (1) patients who were not residents of Puerto Rico at the time of diagnosis, (2) cases from the Veterans Health Administration (VHA) due to institutional restrictions of the VHA, (3) cases without diagnostic confirmation, and (4) cases incorrectly assigned as CLL or AML in the PRCCR database. The study cohort included 926 patients; 518 were patients with CLL, and 408 were patients with AML (see the cohort selection algorithm in Appendix 2).

### 2.4 Outcome variables

To assess the pattern of use of biological and genetic markers for CLL and AML in Puerto Rico, we identified the most relevant genetic and prognostic factors at the time of diagnosis. For CLL cases, we had the FISH test, which is used to identify trisomy 12, del(11q), del(13q), and del(17p). The immunoglobulin heavy chain variable region (IGHV) mutation test was used to identify IGHV mutation status. The status of the TP53 mutation was identified by PCR or FISH. For AML cases, we had information on the karyotype, PCR tests to identify TP53, CEBPA, FLT3, NPM1, c‐Kit, IdH 1&2, and flow cytometry to identify CD33. Furthermore, patients with AML were stratified into risk categories (favorable, intermediate, poor/adverse, not evaluated, and unknown) to determine prognosis and response to treatment, depending on cytogenetic markers^9^ (see the description of selected markers for CLL and AML in Appendix 3).

### 2.5 Independent variables

The factors evaluated in the association of receiving the FISH or PCR test were sociodemographic characteristics at the time of diagnosis, including sex, age group (<50, 50–64, 65–79, ≥80 years), history of previous cancer, type of health insurance (private insurance, Medicaid, Medicare, or dually eligible for Medicare and Medicaid), and the modified Charlson's comorbidity index, classified as 0, 1, ≥2, and unknown comorbidities.^13^, ^14^

### 2.6 Statistical analysis

Descriptive statistics and frequency analyses were used to describe the variables of interest. We used logistic regression models to examine factors associated with the use of the FISH test among patients with CLL and the use of PCR (at least one of c‐Kit, TP53, IDH 1&2, NPM1, FLT3, or CEBPA) among patients with AML. The results of these models are presented in terms of adjusted odds ratios (aOR) and their 95% confidence intervals (CI). Statistical significance was based on two‐sided tests. All analyses were performed using Stata/SE version 15.1 statistical software (Stata Corp.). This study was approved by the Institutional Review Board (IRB) of the University of Puerto Rico Comprehensive Cancer Center (# 2018‐10‐04).

## 3 RESULTS

### 3.1 Characteristics of the cohort by leukemia subtype (CLL and AML)

A total of 926 patients were included in the analysis; of them, 518 had CLL, and 408 had AML. Both leukemia subtypes (CLL and AML) were more common among men and almost half of the patients were between 65 and 79 years old. More patients with AML (22.3%) had previous malignancy than patients with CLL (13.3%), and slightly more than half of patients with CLL and AML had a comorbidity index greater than zero. At the time of diagnosis, 29.3% of patients with CLL were enrolled in Medicare, and 24.3% of patients with AML were enrolled in Medicare‐Medicaid dual insurance (Table 1).

**TABLE 1:** Description of the CLL/AML cohort by leukemia subtype: Puerto Rico, 2011–2015

Characteristics	CLL (*N* = 518)		AML (*N* = 408)	
	Count	%	Count	%
Sex				
Male	306	59.1	208	51.0
Female	212	40.9	200	49.0
Age group (years)				
<50	30	5.8	89	21.8
50–64	135	26.1	92	22.6
65–79	257	49.6	169	41.4
80+	96	18.5	58	14.2
Previous cancer history				
No	449	86.7	317	77.7
Yes	69	13.3	91	22.3
Charlson comorbidity index				
0	233	45.0	190	46.6
1	83	16.0	53	13.0
≥2	91	17.6	67	16.4
Unknown	111	21.4	98	24.0
Insurance at diagnosis				
Private	119	23.0	93	22.8
Medicaid	77	14.9	96	23.5
Medicare/Medicaid	117	22.6	99	24.3
Medicare	152	29.3	84	20.6
Unknown/Other	53	10.2	36	8.8

### 3.2 Prevalence of prognostic markers in CLL and AML

In general, the FISH test was reported in 85.1% of patients with CLL; among these, more than half carried deletion 13q and almost a quarter (24.0%) had the presence of trisomy 12. PCR testing was reported in 83.4% of patients with CLL, of those, 3.2% had the TP53 mutation. Meanwhile, the IGHV test was reported in 60.2% of patients with CLL, of which 57.1% of patients had mutated IGHV (Table 2).

**TABLE 2:** Pattern of use of biological and genetic tests and prevalence of prognostic markers in chronic lymphocytic leukemia

Biological and genetic tests	Total patients with test reported *N* (%)	Prognostic markers	Prevalence *N* (%)
FISH^a^	441 (85.1%)	del(13q)	222 (50.3%)
		Tri12^b^	106 (24.0%)
		del(11q)	42 (9.5%)
		del(17p)	28 (6.4%)
PCR	432 (83.4%)	TP53^c^	14 (3.2%)
IGHV mutation testing	312 (60.2%)	IGHV	178 (57.2%)

^a^ At least one of trisomy 12, deletion 11q, deletion 13q, deletion 17p.

^b^ Trisomy 12 was reported only in 433 cases.

^c^ TP53 is detected using FISH or PCR.

**FIGURE 1:**
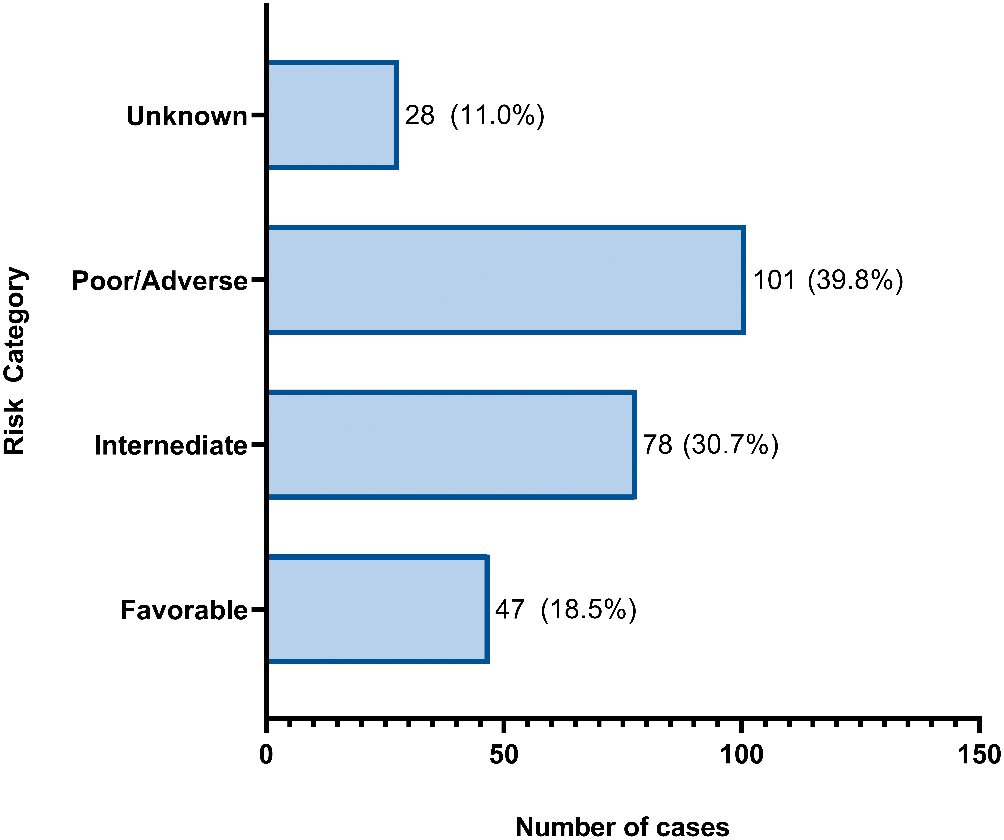
Distribution of risk category for AML cases.

We assigned AML risk categories only among the 254 patients who had reported cytogenetics and molecular tests (Table 3); of these, 18.5% had favorable risk, 30.7% had intermediate risk, 39.8% had poor or adverse risk, and 11.0% had unknown risk (Figure 1). Karyotype was reported in 265 AML patients. Among patients who had undergone karyotype testing, 64.2% had an abnormal karyotype and among those with an abnormal karyotype, 42.4% had a complex karyotype. The c‐Kit test was the highest PCR test reported, and among the patients who had the test, 86.7% had a c‐Kit mutation. Meanwhile, flow cytometry, used to identify CD33 was reported in 297 patients with AML, of whom almost all (92.3%) showed expression of CD33.

**TABLE 3:** Pattern of use of biological and genetic tests and prevalence of prognostic markers in acute myeloid leukemia

Biological and genetic tests	Prognostic markers	Total patients with test reported *N* (%)	Prevalence of prognostic markers *N* (%)
PCR	c‐Kit	308 (75.5%)	267 (86.7%)
	TP53	15 (3.7%)	10 (66.7%)
	IDH 1&2	19 (4.7%)	11 (57.9%)
	NPM1	144 (35.3%)	38 (26.4%)
	FLT3	159 (39.0%)	35 (22.0%)
	CEBPA	144 (35.3%)	21 (14.6%)
Flow cytometry	CD33	297 (72.8%)	274 (92.3%)
Cytogenetic test	Normal karyotype	265 (64.6%)	95 (35.9%)
	Abnormal karyotype^a^		170 (64.2%)
	Complex karyotype		72 (27.2%)

^a^ Includes patients with complex karyotype.

### 3.3 Association between the use of prognostic tests/markers and patients' characteristics

Patients older than 74 years were 61% (aOR = 0.39; 95% CI: 0.23–0.67) less likely to have had a FISH test compared to those younger than 75 years. In addition, patients with a comorbidity index equal to one were 66% (aOR = 0.34; 95% CI: 0.17–0.69) less likely to be given FISH testing compared with those with a comorbidity index equal to zero. Meanwhile, Medicaid patients were less likely to be tested for TP53 compared to other types of insurance (*p* < 0.05) (Table 4). In terms of factors associated with undergoing IGHV testing, the analysis does not show any statistical associations between the different selected predictors and the performance of IGHV testing (*p* > 0.05) (data not shown).

**TABLE 4:** Factors associated with performing FISH test among patients with chronic lymphocytic leukemia and PCR in patients with acute myeloid leukemia

Characteristics	FISH test among patients with CLL			PCR test among patients with AML		
	aOR	95% CI	*p*‐value	aOR	95% CI	*p*‐value
Sex						
Male	1.00			1.00		
Female	1.30	(0.77–2.20)	0.322	1.05	(0.65–1.71)	0.838
Age at dx, years						
<75	1.00			1.00		
≥75	0.39	(0.23–0.67)	0.001	1.81	(0.97–3.38)	0.062
Insurance in diagnosis						
Private	1.00			1.00		
Medicaid	0.54	(0.22–1.32)	0.176	0.84	(0.42–1.69)	0.619
Medicare	1.38	(0.57–3.32)	0.472	0.96	(0.43–2.16)	0.929
Medicare/Medicaid	0.74	(0.32–1.72)	0.485	1.36	(0.61–3.03)	0.450
Others/Unknown	0.38	(0.11–1.33)	0.131	0.51	(0.19–1.39)	0.189
Charlson comorbidity index						
0	1.00			1.00		
1	0.34	(0.17–0.69)	0.003	0.82	(0.38–1.78)	0.621
≥2	0.48	(0.23–1.01)	0.052	1.25	(0.54–2.90)	0.610
Unknown	1.24	(0.47–3.27)	0.660	0.71	(0.37–1.39)	0.322

Abbreviation: aOR, Adjusted Odd Ratio.

When we analyzed factors associated with undergoing PCR among patients with AML, we did not find any significant statistical associations with any of the predictors included in the analysis (*p* > 0.05) (Table 4). Similarly, regarding factors associated with undergoing CD33 testing, the analysis does not show any associations between the different selected predictors and the performance of CD33 testing (*p* > 0.05) (data not shown). Furthermore, among patients older than 74 years, the likelihood of being tested for the karyotype is twice than that of younger patients (aOR = 2.04; 95% CI: 1.20–3.45). Similarly, patients with private insurance were twice as likely to have the karyotype tested compared to Medicaid patients with Medicaid (aOR = 2.06; 95% CI: 1.10–3.85) (data not shown).

## 4 DISCUSSION

In recent decades, the medical management of patients with CLL and AML has improved in diagnosis, prognosis, and monitoring, particularly in understanding genetic markers.^15^, ^16^ Genetic testing is a key tool to evaluate and guide treatment decisions among patients with CLL and AML. To our knowledge, this is the first study to assess the pattern of use and prevalence of biological and genetic markers of CLL and AML among a homogenous Hispanic population. In Puerto Rico, these tests are not performed consistently among patients with CLL and AML; however, the frequency of genetic testing was higher than that reported in other studies.^17^, ^18^ This difference might be attributable to the high coverage of health insurance and the adherence of healthcare professionals in Puerto Rico to recommended evidence‐based treatment guidelines.^19^ However, the amount of testing in this cohort is still far from ideal, particularly today when these tests are required for treatment decisions. Therefore, this study is the first step to continue to monitor the management of CLL and AML among Hispanic populations.

### 4.1 Prognostic markers in CLL

FISH testing to identify genetic abnormalities has proved to be relevant in assessing the prognosis of patients with CLL.^20^ However, consistent with previous studies,^17^, ^18^ the age group (<75 years vs. ≥75 years) was an independent predictor of FISH testing. Our findings show that the older patients with CLL are less likely to undergo FISH testing, which is critical to determine treatment modalities. Furthermore, patients with CLL with a comorbidity index greater than zero were less likely to have FISH testing than those with a comorbidity index equal to zero. Although more research is needed to understand these disparities, this may indicate that physicians tend to assess prognostic factors more in the youngest patients due to better outcomes in this population. Nonetheless, according to National Comprehensive Cancer Network (NCCN) guidelines, the FISH panel is recommended for all CLL patients, regardless of age, comorbidities, or other patient characteristics. Whereas elderly patients are less likely to tolerate intensive regimens, more conservative therapies are considered.^21^ For example, for older and/or comorbid patients, currently approved therapies or clinical trials remain options to improve their quality of life.^21^, ^22^, ^23^, ^24^ Future studies are needed to monitor these patterns, since the evaluation of these markers is essential to determine the long‐term prognosis and treatment of patients, regardless of age and comorbidities. Our results show that, excluding del(11q) and trisomy 12, most abnormalities detected by FISH among patients with CLL were similar to those reported by the scientific literature.^25^, ^26^ In Puerto Rico, del(11q) was found in 9.5% of patients with CLL, which is lower compared to other studies (18%–20%).^25^, ^27^ Del(11q) has been associated with shorter disease progression and survival.^25^ Like previous studies, Trisomy12 was the second most common chromosomal abnormality identified. Trisomy 12 was found in 24.0% of patients with CLL in Puerto Rico, which is higher than in other populations with CLL (14%–16%).^25^, ^28^, ^29^, ^30^ However, a recent study using the Connect CLL Registry reported that Trisomy12 was present in 21% of CLL cases in the US.^31^ Possible reasons for this difference could be attributed to the cohort of patients examined, the methods and probes used, and the number of neoplastic B‐cells in the sample analyzed.^32^ Further research is warranted to understand this pattern since few studies compare genetic abnormalities among different populations.

Furthermore, the scientific literature has suggested that patients with unmutated IGHV have a worse prognosis than those with mutated IGHV; the status of IGHV and the TP53 mutations influence the choice of therapy for patients with CLL.^33^, ^34^, ^35^ IGHV is one of the most important molecular prognostic markers for CLL; however, only 60% of CLL patients had this test in Puerto Rico. Additionally, 57.1% of patients with CLL had a mutated IGHV, which is slightly lower than that reported in other studies (60%–70%).^36^, ^37^ Meanwhile, in our study, among patients with CLL who underwent the PCR test, only 3.2% had a mutation in TP53, compared to other studies reporting between 5% and 12%.^38^, ^39^, ^40^

### 4.2 Prognostic markers in AML

For patients with AML, no statistical association was found between selected predictors and those undergoing PCR testing (TP53, CEBPA, FLT3, NPM1, c‐Kit, IDH 1&2). It is important to consider that not all genetic tests were reported for patients with AML. TP53 and IDH 1&2 were reported in only 15 and 19 patients, respectively. Although these results must be interpreted with caution due to the limited sample size, we found a similar prevalence of CEBPA, FLT3, NPM1, and c‐Kit‐related mutations, as in previous studies.^41^, ^42^, ^43^, ^44^, ^45^ One of the reasons for the reported low testing rate for IDH 1&2 could be that they were first used around 2009, close to the study period.^46^ Presently, the performance of these tests is important because there are new drugs targeting patients with IDH1 and IDH2 mutations.^47^ In our study, the FLT3 mutation was found in 21.9% of cases of AML. This is slightly lower than the 27% reported in a large study by the United Kingdom Medical Research Council.^42^ Additionally, c‐Kit was found to be expressed in 86% of AML cases, which is slightly higher than the 60%–80% reported by Malaise et al.^44^ Among patients with AML whose karyotype was reported, 36% have a normal karyotype. This finding is consistent with other studies that reported a normal karyotype in 40% to 48% of patients with AML.^48^, ^49^, ^50^, ^51^ However, a complex karyotype (≥3 clonal abnormalities) was detected in 27% of the patients who received the test, which is higher than that found in the previous studies.^50^, ^52^, ^53^ A possible explanation for these results is that more than 55% of the patients in this study with AML were 65 years or older. Studies have shown that older patients have more complex karyotypes than younger patients.

### 4.3 Strengths and limitations

This study has some limitations that must be acknowledged. First, we could not evaluate relevant clinical information such as physical examination, blood test results, the Rai's/Binet's staging systems, and other tests such as B2 microglobulin. Second, genetic markers could be underreported for various reasons, including failure to be reported to the PRCCR (PRCCR does not collect this clinical data regularly) or not being documented in the pathology reports.^54^ Third, only 20% of the study cohort has been linked to EMR. This limitation has been documented in other studies that use population‐based registry data and clinical trial data.^49^, ^52^ However, it does not affect the generalizability of our results since most of the information for the scope of this study was obtained from the pathology reports database, which was complete and EMR was only used to complement the dataset. Eventually, we expect more physicians to report to the PRCCR through EMR, improving the completeness of the data, particularly physical examination and blood tests. Despite these limitations, our findings highlight the importance of testing for prognostic genetic markers for all patients with CLL and AML and suggest the need for increased awareness and knowledge regarding the value of this genetic information at the time of diagnosis in Puerto Rico. The database developed for this project proved to be an invaluable resource to characterize and monitor the use of biological and genetic markers for CLL and AML in Puerto Rico and potentially could be modified for other cancer sites.

## 5 CONCLUSIONS

Our findings show the potential of the Puerto Rico CLL/AML Population‐Based Registry database to estimate and assess the pattern of use of these biological markers to guide treatment decisions, monitor outcomes, and improve the management among patients with CLL and AML in Puerto Rico. The recommended genetic tests performed among our cohort of Hispanics are inadequate, raising concerns about the treatment decision among this population. This study adds to the scientific literature on CLL and AML among Hispanic populations and could guide public policy and control efforts for these conditions and related morbidities in this population. More studies are needed to understand these patterns and assess the importance of the characteristics of the physician/health system in the performance of these tests.

## AUTHOR CONTRIBUTIONS

**Karen J Ortiz‐Ortiz:** Conceptualization (equal); data curation (supporting); formal analysis (supporting); funding acquisition (lead); investigation (equal); methodology (equal); supervision (equal); visualization (supporting); writing – original draft (equal). **Carlos Ruben Torres‐Cintrón:** Conceptualization (equal); formal analysis (equal); investigation (equal); methodology (equal); project administration (equal); visualization (equal); writing – original draft (equal). **Tonatiuh Suárez Ramos:** Data curation (equal); formal analysis (equal); investigation (equal); methodology (equal); writing – original draft (equal). **Maira Castañeda‐Avila:** Formal analysis (equal); investigation (equal); methodology (equal); writing – original draft (equal). **Luis Cotto Santana:** Investigation (equal); validation (equal); writing – review and editing (equal). **Guillermo Tortolero‐Luna:** Conceptualization (equal); investigation (equal); project administration (equal); supervision (equal); writing – review and editing (equal).

## FUNDING INFORMATION

Financial support for the study was provided by AbbVie Corp. AbbVie participated in the review and approval of the publication. This study was partially supported by the National Program of Cancer Registries (NPCR), Centers for Disease Control and Prevention (CDC; (award number: NU58DP007164) to the Puerto Rico Central Cancer Registry (PRCCR)).

## CONFLICT OF INTEREST

Dr. Ortiz‐Ortiz and Dr. Tortolero‐Luna reported receiving grants from AbbVie Corp. related to the submitted work and grants from Merck & Co outside of the submitted work. No other disclosures were reported.

## ETHICS STATEMENT

This article does not contain studies with human participants performed by any of the authors. This study was approved by the Institutional Review Board (IRB) of the University of Puerto Rico Comprehensive Cancer Center (# 2018‐10‐04).

## Data Availability

This study includes data from the Puerto Rico Central Cancer Registry (PRCCR). Therefore, data from this study will not be available because of the confidentiality agreement between PRCCR and the authors. Nevertheless, investigators could obtain the data through PRCCR following the confidentiality procedures upon reasonable request. PRCCR data can be requested through the following site: http://www.rcpr.org/Datos‐de‐Cancer/Acceso‐a‐Datos.

## APPENDIX 3 Description of selected markers for CLL and AML

**Table:**

Marker	Description
Chronic lymphocytic leukemia	
del(13q)	13q deletion is associated with a more favorable outcome.^55^
Trisomy 12	This common aberration at the time of diagnosis is associated with intermediate prognostic risk.^28^
del(11q)	11q contains the ATM gene, associated with a least favorable outcome, correlated with non‐mutated IGHV genes.^56^
del(17p)	The genetic abnormality involves the TP53 gene and del(11q), associated with a less favorable outcome, correlated with non‐mutated IGHV genes.^38^
TP53	The tumor‐suppressor protein p53 is associated with a poor prognosis. Mutations of TP53 are also associated with poor prognosis independently of the presence of del(17p). It is the strongest prognostic and predictive marker guiding treatment decisions and is associated with markedly decreased survival and impaired response to chemoimmunotherapy.^38^
IGHV	The immunoglobulin heavy chain variable region (IGHV) is used to determine the risk group in newly diagnosed cases. Mutated IGHV is associated with a more indolent clinical course, while cases with unmutated IGHV genes often behave aggressively.^35^
Acute myeloid leukemia	
Cytogenetics (karyotype)	Important for the diagnosis and classification of AML and some are associated with distinctive clinicopathologic features, have prognostic significance, and/or influence the choice of therapy.^57^
c‐kit	Associated with a poor prognosis.^58^
TP53	Aberrations of TP53 are associated with an exceedingly adverse prognosis. TP53 mutations and deletions that include the TP53 locus have a different prognostic impact, with only mutations but not deletions significantly influencing the survival of these patients.^59^
IdH 1	Occur concurrently with NPM1 mutations, associated with CEBPA and absence of FLT3 abnormalities. It is associated with worse outcomes among intermediate risk diseases.^60^, ^61^
IdH 2	The prognostic effect is inconsistent.^61^
NPM1	One of the most commonly mutated genes is associated with a better risk prognosis.^62^
FLT3	The most frequent genetic alteration and a poor prognostic factor.^63^
CEBPA	Associated with lower relapse rate, improved survival, and a favorable risk category.^64^
CD33	CD33 is an excellent myeloid marker and is commonly used for the diagnosis of AML.^65^
